# Applicability of Composite Magnetic Membranes in Separation Processes of Gaseous and Liquid Mixtures—A Review

**DOI:** 10.3390/membranes13040384

**Published:** 2023-03-28

**Authors:** Łukasz Jakubski, Gabriela Dudek, Roman Turczyn

**Affiliations:** 1 Department of Physical Chemistry and Technology of Polymers, Faculty of Chemistry, Silesian University of Technology, Strzody 9, 44-100 Gliwice, Poland; 2 Centre for Organic and Nanohybrid Electronics, Silesian University of Technology, Konarskiego 22B, 44-100 Gliwice, Poland

**Keywords:** magnetic particles, composite membranes, gaseous separation, pervaporation, filtration, adsorption, electrodialysis, membrane distillation

## Abstract

Recent years have shown a growing interest in the application of membranes exhibiting magnetic properties in various separation processes. The aim of this review is to provide an in-depth overview of magnetic membranes that can be successfully applied for gas separation, pervaporation, ultrafiltration, nanofiltration, adsorption, electrodialysis, and reverse osmosis. Based on the comparison of the efficiency of these separation processes using magnetic and non-magnetic membranes, it has been shown that magnetic particles used as fillers in polymer composite membranes can significantly improve the efficiency of separation of both gaseous and liquid mixtures. This observed separation enhancement is due to the variation of magnetic susceptibility of different molecules and distinct interactions with dispersed magnetic fillers. For gas separation, the most effective magnetic membrane consists of polyimide filled with MQFP-B particles, for which the separation factor (α_rat_ O_2_/N_2_) increased by 211% when compared to the non-magnetic membrane. The same MQFP powder used as a filler in alginate membranes significantly improves water/ethanol separation via pervaporation, reaching a separation factor of 12,271.0. For other separation methods, poly(ethersulfone) nanofiltration membranes filled with ZnFe_2_O_4_@SiO_2_ demonstrated a more than four times increase in water flux when compared to the non-magnetic membranes for water desalination. The information gathered in this article can be used to further improve the separation efficiency of individual processes and to expand the application of magnetic membranes to other branches of industry. Furthermore, this review also highlights the need for further development and theoretical explanation of the role of magnetic forces in separation processes, as well as the potential for extending the concept of magnetic channels to other separation methods, such as pervaporation and ultrafiltration. This article provides valuable insights into the application of magnetic membranes and lays the groundwork for future research and development in this area.

## 1. Introduction

Membrane techniques are commonly used to separate various types of liquid and gaseous mixtures. Membrane-based separation processes involve bringing a gaseous or liquid mixture into contact with a semi-permeable membrane, thus enabling the separation of the components of the mixture [1,2]. Over the last decade, researchers have focused on developing composite membranes containing inorganic or organic particles dispersed into the polymeric matrix [3,4,5,6,7]. One type of such filler is a magnetic filler, which, by interacting with selected molecules penetrating through the membrane, facilitates the transport of one component of the mixture over the other one [8]. It was hypothesised that, in addition to the conventional solution-diffusion mechanism typical for dense polymeric membranes, magnetic membranes provide an additional driving force during the transport of species. Furthermore, the agglomeration of particles in the polymer matrix during the membrane formation process creates magnetic channels in the membrane that moderate the transport rate of molecules interacting with the magnetic field [9].

Both the bare magnetic fillers and the membranes filled with them have been classified as either soft or hard magnetic materials. Soft magnets are easily magnetised and demagnetised and have a high saturation magnetisation value, low coercivity, and high permeability [10]. Hard magnetic materials also exhibit a high saturation magnetisation value and high coercivity but are difficult to magnetise and demagnetise [11]. The main difference between hard and soft magnetic fillers is their ability to produce a so-called stray magnetic field. In the case of soft magnetic particles, this field is eliminated by the structure of many magnetic domains. In contrast, hard magnetic particles can retain their single-domain state even in microscopic grains. Due to the difference in magnetic properties of soft and hard magnets, their behaviour in membranes is also different. Consequently, the separation of liquid and gaseous mixtures runs differently in both cases of magnetic particles. Taking advantage of these differences, it is possible to compare individual membranes in specific applications [10,11]. An excellent review published by Bernard et al. in 2009 [12] well summarised the prior state of the art in the field of membrane gas separation techniques; however, it had not yet included any information about the application of magnetic membranes.

This review focused on the comprehensive description of various magnetic membranes applied in gas separation, pervaporation and other separation techniques, including ultrafiltration, nanofiltration, adsorption, electrodialysis and reverse osmosis. For this purpose, the composition of magnetic membranes, as well as their magnetic, lyophilic and structural properties, are extensively discussed. The separation efficiencies are compared with those for membranes without magnetic properties, and a significant improvement in the separation of gaseous and liquid mixtures due to the varying magnetic properties of the membranes is shown.

## 2. Gas Separation

**Figure 1:**
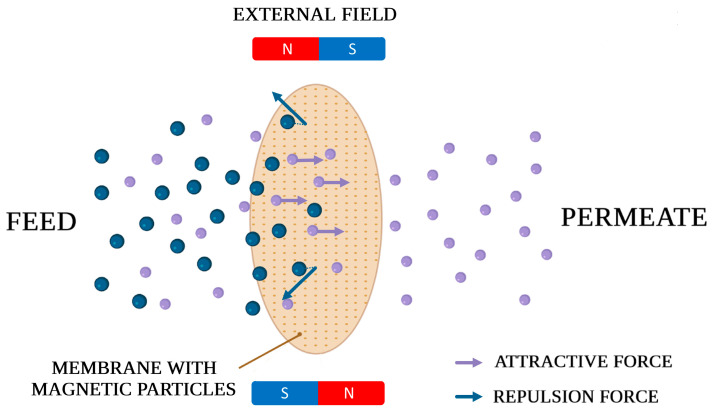
Schematic representation of a separation process undergoing with the use of membrane containing dispersed magnetic particles; arrows indicate magnetic forces acting on gas molecules exhibiting different magnetic properties.

Membranes with magnetic properties have been used to separate oxygen/nitrogen [13,14,15,16,17,18,19,20], carbon dioxide/nitrogen [21,22,23,24], helium/carbon dioxide [25] and carbon dioxide/methane mixtures [23,26]. The magnetic properties of membrane filler and/or additionally applied an external magnetic field impact the separation of gaseous mixtures due to the different magnetic properties of their components as shown in Figure 1. For example, oxygen and nitrogen have been shown to have different magnetic moments and mass magnetic susceptibility. Additionally, oxygen is a paramagnet, and nitrogen is a diamagnet [8]. Other gaseous mixtures have diamagnetic properties, but the difference in magnetic susceptibility allows us to separate them easier under the influence of a magnetic field. Furthermore, the addition of magnetic fillers affects the formation of magnetic channels into the polymer matrix that facilitates the penetration through the membrane of molecules interacting with the magnetic filler [27,28]. In some cases, the magnetic properties of filler enhance its uniform distribution in the polymer matrix, resulting in improved gas separation performance.

The direction and polarity of the magnetic flux produced by the membrane also have a strong effect on the efficiency of gas separation. As shown in the study of Cieśla et al. [29], when using an external magnetic field to direct the magnetic flux, the direction of the magnetic flux produced by the membrane and polarised by the external magnetic field is perpendicular to the direction of the pressure gradient. Therefore, it is possible to achieve an inverse separation effect. In this case, nitrogen molecules are attracted by the membrane and oxygen molecules are repelled. However, this relationship has not been exploited in previous studies of the separation of gaseous mixtures using magnetic membranes due to the difficulties associated with polarising the magnetic field flux in the parallel direction to the membrane when using powdered fillers. In addition, this work suggested that porous superconductors can be used as magnetic separator membranes to separate oxygen and nitrogen. In this case, once the superconducting state is reached, the superconductor turns into a perfect diamagnetic (Meissner effect), and a concentration of magnetic field lines is observed on its surface and in its pores. As a result, a magnetic field gradient is formed. However, the flux of the magnetic field is parallel to the gas pressure gradient so that oxygen molecules are attracted to the membrane while nitrogen molecules are repelled. The published study [14] shows that the mutual position of the magnetic field vectors and the gas flow velocity cannot be neglected for effective gas separation. If these vectors are arranged parallel to the membrane’s surface, the gas separation effect will be opposite to that of a porous superconductor.

The addition of magnetic particles into the polymer matrix impacts the mechanical properties of the obtained membranes [13,15,30]. The value of Young’s modulus depends on whether an external magnetic field was applied at the preparation stage. The study by Rybak et al. [30] showed that membranes subjected to drying without an external magnetic field exhibited poorer mechanical properties than membranes dried in an external magnetic field. In addition, the force of the magnetic field influenced the strength of the membranes in such a way that stronger membranes were obtained in a higher external magnetic field. The improvement in the strength of the hybrid membranes is associated with a decrease in the mobility of the polymer chains, an increase in density, and a more compact structure of the membrane, which is easier to achieve with an external magnetic field. Among all the discussed membranes, the highest value of Young’s modulus (1600 MPa) was noted for poly(p-phenylene oxide) membrane (PPO) containing 5 wt% of iron-decorated carbon nanotubes Fe@MWCNT [30]. Furthermore, it was shown that the application of an external magnetic field during the process of membrane drying impacts the separation properties but only when the soft magnets are used as fillers [9,31]. The soft magnets are magnetised in the presence of a magnetic field, and this magnetisation goes to zero when the magnetic field is removed [32]. Consequently, applying a soft magnet as a filler requires the presence of an external magnetic field not only during the drying of the membrane but also during the separation process of the gaseous mixture. Furthermore, the magnetic properties of membranes increased with the amount of a soft magnet filler. Raveshiyan et al. [9] observed the highest saturation magnetisation value (23 em·g^−1^) for a polysulfone (PSf) membrane modified with 10 wt% carbonyl iron powder. On the other hand, hard magnets have a high coercivity, which makes it difficult to magnetise and demagnetise them [14,17,18,30,33,34]. For this reason, placing the membranes loaded with hard magnets in an external magnetic field is unnecessary to obtain advantageous separation results. Among all studied membranes with a hard magnet as a filler, the best magnetisation results were noted for membranes with an ethyl cellulose (EC) matrix filled with 1.5 g MQFP-14-12 (D50 = 11 μm) and sulfonated poly(2,6-dimethyl-1,4-phenylene oxide) (FeSPPO) matrix filled with 1.8 g MQFP-14-12 (D50 = 7 μm) described by Rybak et al. [18,31] for which the coercivity values reached 960 and 870 kA·m^−1^, respectively.

The addition of magnetic fillers to the polymer matrix significantly affects the separation of gaseous mixtures. For oxygen/nitrogen mixture separation, the permeability of considered molecules increases with the amount of magnetic filler due to the interactions between the magnetic membrane and the gaseous molecules. Furthermore, the permeability of O_2_ molecules is higher than that of N_2_ molecules, which is attributed to the magnetic properties of oxygen and nitrogen. O_2_ is a paramagnetic substance attracted by a strong magnetic field, while N_2_ is a diamagnetic substance repelled by the magnetic field. Additionally, the kinetic diameter of oxygen molecules is smaller compared to the diameter of nitrogen molecules, resulting in an easier permeation of oxygen than nitrogen molecules through membranes.

Raveshtyan et al. [9] studied the permeability of O_2_ and N_2_ molecules through magnetic membranes filled with soft magnets in the presence and absence of an external magnetic field. Without an external magnetic field, a low O_2_/N_2_ selectivity was observed, which was associated with the lack of magnetic properties of soft magnets in the absence of a magnetic field. Therefore, both gases were able to penetrate through the membrane in a similar manner. However, an external magnetic field often entails a more effective separation of oxygen and nitrogen. In the presence of an external magnetic field (B = 570 mT), the PSf membrane containing 10 wt% of carbonyl iron powders (CIPs) in polymer matrix showed an increased selectivity of O_2_/N_2_ by 128% when compared to the separation process performed without an external magnetic field. Moreover, for this type of membrane, oxygen permeability increases with an increase in the force of the external magnetic field, while nitrogen permeability decreases, leading to a rise in membrane selectivity. In the presence of a magnetic field, oxygen molecules are attracted to the membrane, which results from the magnetic interaction between paramagnetic oxygen molecules and soft magnets incorporated into the membrane. In contrast to oxygen, there is a repulsive force between nitrogen molecules and the soft magnets, which prevents the diffusion of nitrogen molecules through the membrane. Thus, by increasing the charge of the particles, the magnetic properties of the membranes are enhanced, as well as the interactions between gas molecules and magnetic particles, as a result of which the effect of the magnetic field is also increased. For membranes containing hard magnets as a filler, the same relationships between the permeability of oxygen and nitrogen molecules through the membranes are observed as for membranes with soft magnets as a filler working in an external magnetic field. In this case, membrane selectivity is directly related to coercivity.

So far, the separation of O_2_/N_2_ mixture based on magnetic membranes has been described for the following systems: ethyl cellulose, poly(2,6-dimethyl-1,4-phenylene oxide) (PPO) and linear (LPI) or hyperbranched (HBPI) polyimide as a polymer matrix filled with various neodymium powders: MQFP-B (Nd-Fe-B alloy), MQFP-C (Nd-Fe-Co-B alloy), MQFP-14-12 (Nd-Fe-Nb-B alloy), MQFP-16-7 (Pr-Fe-Co-Nb-B alloy) [13,14,15,16,17,18,19,20]. For a sulfonated FeSPPO filled with 1.8 g of neodymium MQFP-14-12 powder (D50 = 7 μm), the O_2_/N_2_ selectivity increased by 51% compared with the pristine membrane [31]. Additionally, in polyimide hollow fibre membranes with γ-Fe_2_O_3_ nanoparticles (PHFM) and pyrolysed ones (CHFM) [33], the carbonisation of polyimide hollow fibre membranes increased the ideal separation coefficient significantly, but with a parallel 1000-fold decrease in permeation rate. During pyrolysis in an inert atmosphere, γ-Fe_2_O_3_ nanoparticles were transformed into Fe_3_C. PSf membranes filled with CIPs and neodymium [9,34], sulfonated poly(2,6-dimethyl-1,4-phenylene oxide) (SPPO) membranes modified by the addition of sodium or iron(II) ions, and iron-encapsulated multi-wall carbon nanotubes (Fe@MWCNTs) [30], poly(ethersulfone) (PES) membranes filled with iron-nickel Fe_10_Ni_90_ and Fe_20_Ni_80_ particles [35] were also considered in the process of oxygen/nitrogen mixture separation. In all these cases, scientists used filler powders with various granulation and different concentrations. The fillers were added to the polymer solutions and dispersed during sonication. After that, the mixtures were poured into Petri dishes. Moreover, in some papers, the external magnetic field was applied during membrane drying to arrange the alignment of the particles along the magnetic field. The mass transport coefficients of pure oxygen, nitrogen, and O_2_ and N_2_ in the air for various magnetic membranes are collected in Table 1.

The magnetic membranes were also applied for the separation of the CO_2_/N_2_ mixture. For instance, membranes with a polyethylene glycol (PEG) matrix incorporated with iron dopamine nanoparticles (FeDA NPs) [21], poly(amide-6-b-ethylene oxide) [PEBA] with an iron benzene-1,3,5-tricarboxylate (Fe-BTC) filler [22], [PEBA] filled with Fe_2_O_3_ nanoparticles [23], magnetic liquid membranes based on [P_6,6,6,14_]^2+^[CoCl_4_]^2−^, [P_6,6,6,14_]^+^[FeCl_4_]^−^, [P_6,6,6,14_]^2+^[MnCl_4_]^2−^ and [P_6,6,6,14_]^3+^[GdCl_6_]^3−^ [24], supported with a PVDF were investigated. The mass transport coefficients of CO_2_ and N_2_ for these magnetic membranes are collected in Table 2. In the case of the He/CO_2_ mixture, a successful separation was achieved by using binary iron oxide/cobalt oxide silica membranes [25], and for the CO_2_/CH_4_ mixture, PEBA filled with Fe_2_O_3_ nanoparticles [23] and poly(2,6-dimethyl-1,4-phenylene) oxide (PPO_dm_) membrane incorporated with α-Fe_2_O_3_/TiO_2_ magnetic filler [26] were studied.

Although both N_2_ and CO_2_ exhibit diamagnetic properties, the magnetic susceptibility of CO_2_ is stronger than that of N_2_ molecules, causing the possibility of separating them effectively in a magnetic field. Consequently, N_2_ molecules are more strongly repelled from the magnetic field [36], and the permeability of CO_2_ molecules is higher than that of N_2_ molecules [21,22,30,35]. Furthermore, the permeability of CO_2_ molecules depends on the amount of magnetic filler and is larger for higher filler content. In the case of PEBA membranes filled with Fe_2_O_3_ particles, the increase in permeability compared to the pristine membrane was equal to around 154 %. For PEBA with Fe-BTC filler, the increase was equal to 49 %, while for the PEG matrix with FeDA NPs, the permeability increased by as much as 210 %. In addition, the increase in permeability of CO_2_ molecules contributed directly to the increase in selectivity as the magnetic strength of the membrane increased. However, Albo et al. [24] studied another type of magnetic membrane, namely magnetic liquid membrane, and showed that the membrane based on [P_6_,_6_,_6_,_14_]^2+^[MnCl_4_]^2−^ was effective in the CO_2_/N_2_ mixture separation.

Helium and carbon dioxide molecules also exhibit diamagnetic properties but differ in magnetic susceptibility. Darmawan et al. [25] showed that combined binary iron oxide/cobalt oxide silica membranes could greatly intensify the He/CO_2_ separation. In this case, the permeability of helium molecules was much higher than CO_2_ molecules for all the modified membranes used, which was associated not only with the difference in the reaction of the molecules with the magnetic field but also with the difference in the size of the kinetic diameters of CO_2_ and He. The researchers achieved magnetic membrane selectivity of He/CO_2_ of 135.00, which means that the separation was greatly intensified.

The application of a magnetic field in the process of gaseous separation was also investigated for a CH_4_/CO_2_ mixture. Harami et al. [23] applied PEBA membranes filled with Fe_2_O_3_ nanoparticles, whereas Yap et al. [26] studied PPO_dm_ membrane incorporated with α-Fe_2_O_3_/TiO_2_ magnetic filler. For these membranes, the permeability of CH_4_ was much higher than that of CO_2_, which was explained by the difference in the interaction of individual molecules with the magnetic field generated into the membrane. CH_4_ molecules interact more easily with the magnetic field and are more easily attracted by it, so the permeability of CH_4_ molecules is higher than that of CO_2_ molecules. The results of the separation processes showed that PEBA and PPO_dm_ membranes filled with magnetic particles increase CH_4_/CO_2_ selectivity by 100% and 42%, respectively, when compared to non-magnetic membranes.

To summarise, magnetic membranes improve gas separation performance due to the interaction of magnetic particles with molecules penetrating a polymer matrix. These particles can enhance the diffusion of gas molecules, leading to higher separation efficiency. Moreover, external magnetic fields can induce additional forces on the gas molecules within the membrane, further improving the selectivity and permeability of the separation process. This feature can be particularly advantageous for the separation of complex gas mixtures or in applications requiring precise control over the separation process. The advantages of magnetic membranes have been demonstrated in various studies and applications. These examples highlight the potential of magnetic membranes as promising gas separation technology with various practical applications and as a viable alternative to traditional non-magnetic membranes for gas separation.

## 3. Pervaporation

Pervaporation is a similar process to gas permeation, but the main difference is another phase composition of the feed solution–here, it is a liquid mixture. The components of the permeate are vaporised in a vacuum or inert carrier gas on the “low pressure” side of the membrane. The technique uses non-porous asymmetric and composite membranes made of glassy or flexible polymers. So far, magnetic membranes have been applied only in the case of the ethanol/water mixture separation process. The reason for using magnetic membranes in the separation of water/ethanol mixture is due to the difference in polarity and dipole moment values of water and ethanol molecules. Water is a dipole, so it interacts strongly with magnetic substances, while ethanol is less affected by magnetic fields due to its lower polarity [37].

As a matrix of magnetic membranes used in ethanol dehydration via pervaporation, sodium alginate, chitosan and poly(vinyl alcohol) were investigated [38,39,40]. The alginate membranes were filled with MnO_2_, MnO_2_@Fe_3_O_4_, Fe_3_O_4_, Fe_3_O_4_@CNT, FexN/CXG, Fe_2_O_3_, Fe(acac)_3_, Fe_4_(acac)_6_(Br-mp)_2_, Fe_4_[Fe(CN)_6_]_3_, MQFP, ZnO, Ag_2_O, TiO_2_, Cr_2_O_3_ magnetic particles [38,39,40,41,42,43,44,45]. For chitosan membranes, TiO_2_, Cr_2_O_3_, Fe_3_O_4_, Fe_4_[Fe(CN)_6_]_3_ and for poly(vinyl alcohol) (PVA) membranes Fe_3_O_4_, Fe_2_O_3_, Fe(acac)_3_ were used as a magnetic filler [38,39,40,41,42,43,44,46,47,48,49]. The membranes made by combining chitosan/polysulfone and PVDF-HFP/GO/ODS composite hollow fibre were also tested [50]. Unfortunately, the authors did not report the magnetic properties of the used HFP/GO/ODS filler. Magnetic membranes applied in ethanol dehydration via pervaporation can be prepared in two ways. The first and most popular is the casting and solvent evaporation method used to make membranes with all the mentioned matrices. However, Gao et al. [45] and Zhao et al. [51] designed the composite alginate membranes by spin-coating a blend of sodium alginate solution containing Fe_3_O_4_@CNT or Fe_3_O_4_ nanofillers onto polyacrylonitrile ultrathin membranes. In all cases, the membranes were cross-linked with agents that contributed to better separation due to the restriction of membrane extensive swelling. Thus, for alginate membranes, calcium dichloride was used as a cross-linking agent. For PVA membranes, glutaraldehyde, while for chitosan membranes, sulphuric acid or glycidyl chloride in sodium hydroxide solution were used as cross-linking agents.

The separation process of water/ethanol molecules through such membranes was possible due to the different interactions of water and ethanol molecules with a magnetic filler, e.g., by the interaction with magnetic channels [27] and the hydrophilic properties of investigated membranes. According to Wang et al. [52], the magnetic field affects the physicochemical properties of water, e.g., speeds up its evaporation, reduces the friction coefficient and consequently promotes a faster permeation of water through the membrane. On the other hand, water/ethanol separation processes are possible using magnetic membranes mainly because of the differences in polarity and magnetic properties of water and ethanol, causing the occurrence of different interactions of molecules with a magnetic field created by magnetic particles. Having a structure of a dipole, permeating water molecules interact with magnetic substances present in a membrane, leading to accelerated water penetration. Because of the less polar structure of ethanol, the magnetic field has not the same effect on ethanol as on water [53]. The studies of Gao et al. [45] and Dudek et al. [38] showed that magnetic powders used as fillers for membranes applicable in pervaporation processes were more or less hydrophilic, impacting the lyophilic properties of magnetic membranes. The most hydrophilic membranes with the lowest contact angle and the highest degree of swelling were obtained for the alginate matrices filled with Fe_3_O_4_@CNT particles on the polyacrylonitrile porous support layer [45] and for the PVA matrices filled with 15 wt% Fe_2_O_3_ particles [38]. For these two systems, the contact angle and degree of swelling values were very similar, i.e., 26° and 260 % for SA-Fe_3_O_4_@CNT and 28° and 250 % for PV15 Fe_2_O_3_, respectively.

The critical parameter that impacts the separation of the water/ethanol mixture through magnetic membranes is the dispersion of magnetic particles into the polymer matrix. The rate of dispersion depends on the magnetic properties of investigated fillers, i.e., whether it is a soft or a hard magnet. The following species: Fe_3_O_4_, Fe_3_O_4_@CNT, Fe_x_N/C_XG_, ZnO, Ag_2_O, TiO_2_, and Cr_2_O_3_ showed limited ferromagnetic properties [39,40,41,44,45,47,53] and could be not quite uniformly dispersed, often forming visible agglomerates in the polymer matrix. The exception was MQFP powder used as a filler, especially a fine grounded one, which had good compatibility with the membrane material. Weak dispersion of magnetic fillers was observed by Dudek et al. [38], who used antiferromagnetic Fe_2_O_3_ as a filler. This problem, however, was not observed in the case of membranes filled with paramagnetic molecular magnets and iron complexes. Their excellent dispersion in polymer matrices made it possible to formulate membranes with superparamagnetic properties. These fillers include: Fe(acac)_3_, Fe_4_(acac)_6_(Br-mp)_2_, and Fe_4_[Fe(CN)_6_]_3_ [38,42].

The addition of magnetic filler to the polymer matrix significantly affected the separation efficiency of the water/ethanol mixture commonly expressed by separation factor (α) and Pervaporation Separation Index (PSI) [54]. The first one determines the ability of a membrane to selectively separate a two-component feed solution. On the other hand, PSI seems to be more universal since it combines both the value of the permeate flux (speed of separation) and the separation factor (separation effectiveness). Therefore, it is possible to more accurately assess the performance and separation efficiency of the feed by different membranes. The results presented by Dudek et al. [42] showed that the most important parameters impacting the separation efficiency of the water/ethanol mixture were the dispersion efficiency and character of the magnetic filler. According to the already published studies, the membrane with the best pervaporation efficiency for ethanol dehydration is an alginate membrane filled with 1 wt% of MQFP powder with D50 = 15 µm, which was investigated by Grzybek et al. [39]. The PSI and α values for this membrane reached 30,457 kg∙m^−2^∙s^−1^ and 12,271, respectively. At this time, this membrane is unrivalled, as it shows almost seven times higher pervaporation efficiency than the second-best membrane investigated by Gao et al. (PSI = 4130.5 kg∙m^−2^∙s^−1^ and α = 1870) [45]. Such good results were attributed to the use of a relatively hard magnet MQFP filler and its uniform dispersion in a polymer matrix. In this case, the MQFP particles did not cluster into domains because of their good compatibility with the alginate matrix. Because of their even distribution, they formed a regular magnetic field inside the membrane without the gaps, through which the particles could permeate without interacting with magnets. This close contact between magnets and the permeating molecules seems to be the necessary factor in achieving a high separation efficiency. Dudek et al. [47,48] studied the influence of the content of soft ferromagnetic Fe_3_O_4_ particles loaded into alginate and chitosan matrixes on water/ethanol separation effectiveness. The highest value of PSI (76.6 kg∙m^−2^∙s^−1^) was achieved for the alginate membrane containing 15 wt% of magnetite, and such value was more than 50 times lower than for the same matrix filled with hard magnet MQFP powder. One of the reasons for the lower efficiency of Fe_3_O_4_-loaded membranes was the weak dispersion of ferromagnetic particles in the alginate matrix. On the other hand, soft magnets in the form of iron complexes Fe(acac)_3_ and Fe_4_(acac)_6_(Br-mp)_2_ loaded into PVA and alginate membranes showed homogeneous dispersibility in the membrane but using only the Fe(acac)_3_ filler the efficiency of ethanol dehydration was not satisfactory: the PSI reached only 21.0 kg∙m^−2^∙s^−1^ with the separation factor equal to 11.8. However, when Fe_4_(acac)_6_(Br-mp)_2_ was applied as a filler of the alginate matrix, the PSI value reached 1275.5 kg∙m^−2^∙s^−1^ and the separation factor increased by 485 % compared to the membrane filled with Fe(acac)_3_ particles [38,42]. The combined powder was also used as a magnetic filler in an alginate matrix by Grzybek et al. [43], who used an in situ precipitated MnO_2_@Fe_3_O_4_ to achieve a synergistic effect of these two oxides with different magnetic characteristics. The results showed that the combination of fillers led to better results than if they were applied separately. The PSI of such membrane increased more than seven times if compared with analogues membrane filled only with a plain Fe_3_O_4_ and reached the value of 588.0 kg∙m^−2^∙s^−1^. The surface modification of Fe_3_O_4_ with an in situ precipitated MnO_2_ overcame the typical challenges of using magnetic particles alone, that is, to achieve adequate dispersion and stabilisation of Fe_3_O_4_ nanoparticles in the polymer matrix. This approach appears promising for future research of novel magnetic membrane fillers for application in pervaporation processes. The separation efficiency expressed by separation factor and Pervaporation Separation Index of several magnetic membrane systems in ethanol dehydration are collected in the Table 3.

In summary, this chapter showed the properties and potential applications of magnetic membranes compared to non-magnetic membranes for a pervaporation process. Magnetic membranes offer unique advantages due to their magnetic properties for separating molecules differently interacting with the magnetic field. These properties make magnetic membranes particularly promising for separation processes, such as pervaporation, where they have shown high selectivity and permeability for organic solvents.

## 4. Other Separation Techniques

**Figure 2:**
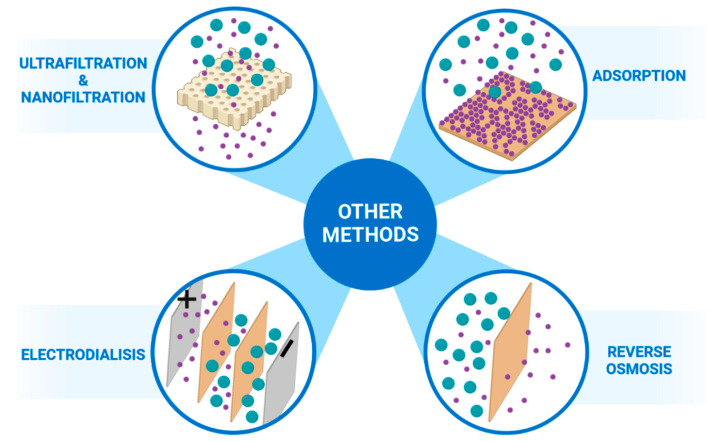
The application of magnetic membranes in other separation methods: ultrafiltration & nanofiltration, for water desalination, adsorptio of cations from water, electrodialysis, reverse osmosis groundwater purification. Created with BioRender.com.

Magnetic membranes were also applied in other processes such as ultrafiltration, nanofiltration, adsorptive separation, electrodialysis and reverse osmosis (Figure 2). As a polymer matrix, mainly poly(vinyl chloride) (PVC), polyethersulfone, polyamide, chitosan and polyamide were used. As a magnetic filler, modified carbon nanotubes Fe_3_O_4_@MWCNT; silica ZnFe_2_O_4_@SiO_2_; oxides Fe_2_O_3_, CoFe_2_O_4_, TiO_2_ and magnetic inorganic-organic hybrid membrane system HMG@MHMG were applied [55,56,57,58,59,60,61,62]. Magnetic membranes used in such processes were most often prepared by casting and a phase inversion method. In general, functionalised magnetic particles are an effective additive to the polymer matrix to prepare hybrid magnetic composite membranes for different applications. All investigated magnetic membranes in other techniques than gas separation and pervaporation showed better separation efficiency than the same without magnetic filler. Liu et al. [55] prepared PVC membranes filled with Fe_3_O_4_@MWCNT particles by a phase inversion method and applied them for a cross-flow filtration of bovine serum albumin from aqueous solutions. They took advantage of the fact that water is strongly attracted to a magnetic field. The flux of pure water reached 487.0 kg∙m^−2^∙h^−1^, and it was nearly doubled compared to the non-magnetic membrane. In contrast, the rejection values increased by 117 %. The same technique was used by Daraei et al. [56] to produce a polyethersulfone (PES) membrane with a similar CNT filler applied for the separation of proteins from an aqueous solution. Daraei et al. have also observed the positive effects of hard magnets in their research. They showed that the flux of pure water reached 65.0, which is 97 % higher than the result obtained for the pristine membrane. Zinadini et al. [57] prepared PES nanofiltration membranes filled with a soft magnet ZnFe_2_O_4_@SiO_2_ for water desalination. The separation results were better for membranes containing 1 wt% of soft magnet nanoparticles for which the water flux reached 34.4 kg∙m^−2^∙h^−1^. Compared to the unfilled membrane, the obtained results are increased by more than four times, significantly boosting the efficiency of desalination. Zinadini et al. associated this phenomenon with a much higher magnetic moment of water than the other species being in a feed. Thus, in the magnetic field created by the membranes, the molecules of water were preferably attracted, which resulted in a significant increase in flux and a large volume of pure water production. Al-Hobaib et al. [58] have incorporated soft magnets as the fillers in composite membranes applied in groundwater purification. They studied the polyamide membranes (TFC) filled with a Fe_2_O_3_ powder in reverse osmosis to groundwater purification process and showed that the flux of purified water reached 44.0 kg∙m^−2^∙h^−1^ and it was 63% higher than for membranes that did not include magnetic filler. However, da Costa Cunha et al. [59], after casting the solution of prepared PES membranes with HMG@MHMG particles, used the solvent evaporation method to obtain a dry membrane for purifying oil-polluted water. Hosseini et al. [60] and Khan et al. [61] also used an evaporation method to prepare PVC membranes filled with CoFe_2_O_4_ particles and chitosan membranes with nickel particles, respectively. Both membranes were used in water deionisation, the first by electrodialysis and the second by adsorptive separation. In the study of Hosseini et al. [60], the permselectivity value for membranes with hard magnet properties was equal to 0.999, whereas for pristine membranes, it reached only 0.958, and the difference was expected to arise from an increase in ion-exchange capacity and an improvement in the membrane’s surface charge density. On the other hand, electrospinning was used by Bassyouni et al. [62] to prepare hybrid composite membranes based on the polyamide 6 fibres through a single-step process by adding Fe_3_O_4_@o-MWCNT particles as a filler. This membrane was successfully used for the adsorptive separation of toxic pollutants in the form of Pb(II) ions from water. The use of magnetic membranes increased the efficiency of the process with a resultant adsorption capacity value reaching 49.3 mg∙g^−1^, making this membrane valuable in industrial applications for wastewater treatment [62].

In the case of membranes filled with hard magnets, the surface charge of the membrane affected the distribution of ions at the solution/membrane interface, which allowed the membrane permselectivity to be tightly controlled and increased. Compared to gas separation and pervaporation, other separation methods have not yet used a combination of fillers exhibiting the properties of hard and soft magnets. It seems like an interesting new idea for the following researchers to look at the results of such a combination for previous applications. Perhaps using this approach, it would be possible to achieve even better results in eliminating the problems caused by soft and hard magnet fillers applied separately.

## 5. Concluding Remarks and Prospects

The application of magnetic membranes is a promising alternative for improving separation efficiency in different separation processes. This review has shown several promising results on the application of magnetic membranes filled with different fillers giving the magnetic character and/or supported also by an external magnetic field in separation processes. It is noteworthy to mention the membranes that have demonstrated the most effective improvement in separation processes. Specifically, for gas separation, the polyimide membrane was filled with MQFP-B particles, for which the separation factor (α_rat_ O_2_/N_2_) increased by 211% compared to the non-magnetic membrane. Additionally, the alginate membrane filled with MQFP powder showed significant improvement in water/ethanol separation via pervaporation, reaching a separation factor of 12,271. For other separation methods, the poly(ethersulfone) nanofiltration membranes filled with ZnFe_2_O_4_@SiO_2_ demonstrated a more than four times increase in water flux compared to the non-magnetic membranes for water desalination. However, the application of magnetic materials for the separation of gaseous and liquid mixtures is still not a common approach. In light of the presented results, it can be concluded that magnetic fillers can desirably impact separation efficiency. Although separated molecules rarely differ significantly in their magnetic properties, even a slight change in *magnetic moments, mass magnetic susceptibility,* or magnetic field flux gives the opportunity to apply the magnetism and magnetic phenomena as an extra force influencing the separation process. However, the proof-of-concept and theoretical explanation of the role of magnetic forces in separation have still not been fully explained. Except for two earlier works by Borys et al. [27] and Cieśla [29], there are no new complete works in this field. The concept of a magnetic channel proposed by Borys et al. for gaseous O_2_/N_2_ separation needs to be further developed and strengthened to better explain the mechanism of overcoming of the Brownian chaotic movement of molecules in gas [27]. Moreover, it should also be extended to other separation methods like pervaporation or ultrafiltration that also benefit from the magnetic-assisted separation.

This review presents an overview of the recent research studies involving the separation techniques in which magnetic membranes have been applied, as well as the separated molecules, investigated polymers matrixes and various magnetic particles. The development of efficient membranes with high flux and selectivity is still challenging. The advantageous effect of magnetic fillers is clearly observed by comparing the separation efficiency of processes conducted with and without magnetic field application. Furthermore, the separation properties depend on the kind of magnetic filler, i.e., soft or hard magnet. The studies showed that the soft magnets could be dispersed into the polymer matrix more uniformly than hard magnets, consequently improving the separation efficiency. On the other hand, the significantly better magnetic properties of hard magnets impact the interactions between the magnetic field and separated molecules. Considering the advantages of both magnetic fillers, some researchers suggest adding a mixed filler, consisting of a soft and a hard magnet, to the polymer matrix. Due to synergistic effects between both magnets, such a filler has proven to be a good solution to eliminate the problems of each filler.

Nevertheless, further systematic investigations on the relations between filler’s magnetic characteristics and membrane structure separation ability are still needed. As most of the research was carried out on a lab scale, future studies need to focus on the larger scale pilot studies to develop highly efficient and resistant membranes, upscaling the process that gives a chance to transfer them to the industrial practice.

In conclusion, magnetic membranes have shown promising results in various separation processes, and the future prospects for this technology are bright. The optimisation of magnetic fillers, investigation of new membrane materials and structures, and upscaling of the process to an industrial scale are areas of future research that could lead to further improvements in separation efficiency. Providing continuous research and development, magnetic membranes have the potential to revolutionise separation processes and contribute to the development of new and innovative separation technologies.

## Figures and Tables

**Table 1:** Permeability (P) and selectivity coefficients (α) determined for pure oxygen and nitrogen, as well as in air for various magnetic membranes.

Membrane		Permeation and Selectivity Coefficients Determined in						References
Matrix	Additive	Pure Gases			Air			
		P_O2_ [Barrer]	P_N2_ [Barrer]	α_id_ O_2_/N_2_	P_O2_ [Barrer]	P_N2_ [Barrer]	α_rat_ O_2_/N_2_	
PSf	CIP	8.47	1.25	6.78	-	-	-	[9]
PSf	Nd	2.05	0.42	4.88	-	-	-	[34]
PHFM	-	2.8 *	1.26 *	2.22	-	-	-	[33]
PHFM	γ-Fe_2_O_3_	1.9 *	1.66 *	1.14	-	-	-	[33]
CHFM	-	7.2 *	0.9 *	8.0	-	-	-	[33]
CHFM	θ-Fe_3_C	8.5·10^−3^ *	0.6·10^−3^ *	14.17	-	-	-	[33]
SPPO	MQFP-14-12	100.26	12.59	7.96	171.73	21.49	7.99	[31]
SPPO	Fe@MWCNT	12.88	2.14	6.03	13.45	2.61	5.15	[30]
HBPI	MQFP-B	90.93	15.58	7.23	232.41	26.75	8.69	[13]
LPI	MQFP-B	29.52	4.00	7.38	70.75	9.51	7.44	[14]
EC	MQFP-16-7	-	-	-	187.70	38.00	4.94	[15]
PPO	MQFP-16	185.30	37.84	4.90	281.96	64.73	4.36	[17,18]
EC	MQFP-14	111.62	45.48	2.44	21.94	3.55	6.18	[20]
PPO	MQFP-14	202.49	40.99	4.94	306.92	68.89	4.56	[19]

* In GPU.

**Table 2:** Permeability (P) and selectivity coefficients (α) determined for the CO_2_/N_2_, He/CO_2_ and CO_2_/CH_4_ mixtures.

Membrane		Permeation and Selectivity Coefficients			References
Matrix	Additive	P_CO2_ [Barrer]	P_N2_ [Barrer]	α_rat_ CO_2_/N_2_	
PEG	FeDA	1265 *	84 *	15	[21]
PEBA	Fe-BTC	92.0	1.9	48.4	[22]
PEBA	Fe_2_O_3_	165.60	1.05	157.71	[23]
PVDF	[P_6_,_6_,_6_,_14_]^2+^ [MnCl_4_]^2−^	202.63	4.92	41.18	[24]
		P_CO2_	P_He_	α_rat_ He/CO_2_	
Silica	Fe/Co oxides	0.36 *	47.76 *	130	[25]
		P_CO2_	P_CH4_	α_rat_ CO_2_/CH_4_	
PEBA	Fe_2_O_3_	165.60	2.96	55.95	[23]
PPO_dm_	α-Fe_2_O_3_/TiO_2_	527.36	126.32	3.43	[26]

* In GPU.

**Table 3:** Comparison of various magnetic membranes efficiency in pervaporative water/ethanol separation process.

Membrane		Separation Factor α _H2O/EtOH_	Pervaporation Separation Index PSI [kg m^−2^ s^−1^]	Reference
Matrix	Additive			
Alginate	MQFP	12,271.0	30,457.0	[39]
Alginate	MnO_2_@Fe_3_O_4_	483.0	588.0	[43]
Alginate	Fe_4_(acac)_6_(Br-mp)_2_	69.0	1275.5	[42]
PVA	Fe(acac)_3_	11.8	21.0	[38]
Alginate	Fe_x_N/C_XG_	65.4	91.5	[44]
Alginate	Fe_3_O_4_	59.0	76.6	[48]
Alginate	Fe_3_O_4_@CNT	1870.0	4130.5	[45]
Chitosan	Fe_3_O_4_	16.3	12.9	[47]
Alginate/PAA	Fe_3_O_4_	1634.0	2661.8	[51]

## Data Availability

No new data were created or analyzed in this study.
